# Incidence, clinical spectrum, diagnostic features, treatment and predictors of paradoxical reactions during antibiotic treatment of *Mycobacterium ulcerans* infections

**DOI:** 10.1186/1471-2334-13-416

**Published:** 2013-09-05

**Authors:** Daniel P O’Brien, Mike Robson, N Deborah Friedman, Aaron Walton, Anthony McDonald, Peter Callan, Andrew Hughes, Richard Rahdon, Eugene Athan

**Affiliations:** 1 Department of Infectious Diseases, Barwon Health, Geelong, Australia; 2 Department of Medicine and Infectious Diseases, Royal Melbourne Hospital, University of Melbourne, Melbourne, Australia; 3 Manson Unit, Médecins Sans Frontières, London, United Kingdom; 4 Department of Pathology, Pathcare, Geelong, Australia; 5 Department of Plastic Surgery, Barwon Health, Geelong, Australia

**Keywords:** Mycobacterium ulcerans, Paradoxical reactions, Antibiotics, Predictors, Incidence, Clinical, Diagnosis, Treatment

## Abstract

**Background:**

Paradoxical reactions from antibiotic treatment of *Mycobacterium ulcerans* have recently been recognized. Data is lacking regarding their incidence, clinical and diagnostic features, treatment, outcomes and risk factors in an Australian population.

**Methods:**

Data was collected prospectively on all confirmed cases of *M. ulcerans* infection managed at Barwon Health Services, Australia, from 1/1/1998-31/12/2011. Paradoxical reactions were defined on clinical and histological criteria and cases were determined by retrospectively reviewing the clinical history and histology of excised lesions. A Poisson regression model was used to examine associations with paradoxical reactions.

**Results:**

Thirty-two of 156 (21%) patients developed paradoxical reactions a median 39 days (IQR 20-73 days) from antibiotic initiation. Forty-two paradoxical episodes occurred with 26 (81%) patients experiencing one and 6 (19%) multiple episodes. Thirty-two (76%) episodes occurred during antibiotic treatment and 10 (24%) episodes occurred a median 37 days after antibiotic treatment. The reaction site involved the original lesion (wound) in 23 (55%), was separate to but within 3 cm of the original lesion (local) in 11 (26%) and was more than 3 cm from the original lesion (distant) in 8 (19%) episodes. Mycobacterial cultures were negative in 33/33 (100%) paradoxical episodes. Post-February 2009 treatment involved more cases with no antibiotic modifications (12/15 compared with 11/27, OR 5.82, 95% CI 1.12-34.07, p = 0.02) and no further surgery (9/15 compared with 2/27, OR 18.75, 95% CI 2.62-172.73, p < 0.001). Six severe cases received prednisone with marked clinical improvement. On multivariable analysis, age ≥ 60 years (RR 2.84, 95% CI 1.12-7.17, p = 0.03), an oedematous lesion (RR 3.44, 95% CI 1.11-10.70, p=0.03) and use of amikacin in the initial antibiotic regimen (RR 6.33, 95% CI 2.09-19.18, p < 0.01) were associated with an increased incidence of paradoxical reactions.

**Conclusions:**

Paradoxical reactions occur frequently during or after antibiotic treatment of *M. ulcerans* infections in an Australian population and may be increased in older adults, oedematous disease forms, and in those treated with amikacin. Recognition of paradoxical reactions led to changes in management with less surgery, fewer antibiotic modifications and use of prednisolone for severe reactions.

## Background

*Mycobacterium ulcerans* causes necrotizing lesions of skin and subcutaneous tissue [1]. The necrotic lesions are characterized by minimal associated inflammation thought to result from immunomodulatory effects of its pathogenic toxin mycolactone which impairs both local and systemic immune responses to the infection [2,3].

Antibiotics are now recommended as initial treatment for *M. ulcerans* infections [4,5]. Recently paradoxical reactions, or immune reconstitution inflammatory syndrome reactions, have been recognized to occur following antibiotic treatment, [6-9] and can be mistaken for treatment failure. Although more research needs to be done to clarify the aetiology of paradoxical reactions, it is proposed their pathogenesis may involve reversal of an immune-inhibitory state induced by mycolactone, [10-12] due to antibiotic mediated killing of the organism and the secondary reduction in mycolactone levels [13-15]. This enables the development of an intense immunological reaction presumably against persisting dead or viable mycobacteria, [6,8,15] manifest clinically by worsening of existing, or the appearance of new, lesions and histologically by the appearance in lesions of intense inflammation [6,15].

Currently data in an Australian population is lacking regarding the incidence, clinical and diagnostic features, treatment, outcomes and predictors of patients developing paradoxical reactions associated with antibiotic treatment of *M. ulcerans*. In this study we describe these factors amongst a patient cohort treated for *M. ulcerans* infections acquired in the Bellarine Peninsula, south-eastern Australia.

## Methods

Data on all confirmed *M. ulcerans* cases treated at Barwon Health were collected prospectively from 1/1/1998-31/12/2011. Cases of paradoxical reactions were determined by retrospectively reviewing the clinical history and histology of excised lesions.

A *M. ulcerans* case was defined by a lesion clinically suggestive of *M. ulcerans* plus any of (1) a culture of *M. ulcerans* from the lesion, (2) a positive polymerase chain reaction (PCR) test from a swab or biopsy of the lesion, or (3) histopathology of an excised lesion showing a necrotic granulomatous lesion with the presence of acid-fast bacilli (AFB) consistent with acute *M. ulcerans* infection.

Paradoxical reactions were defined by the presence of one or both of the following features: a) clinical: an initial improvement on antibiotic treatment in the clinical appearance of a *M. ulcerans* lesion followed by deterioration of the lesion or its surrounding tissues, or the appearance of a new lesion(s), and b) histopathology: examination of excised tissue from the clinical lesion showing evidence of an intense inflammatory reaction consistent with a paradoxical reaction [6]. A ‘severe’ paradoxical reaction was a clinical assessment determined by the treating clinician.

**Figure 1:**
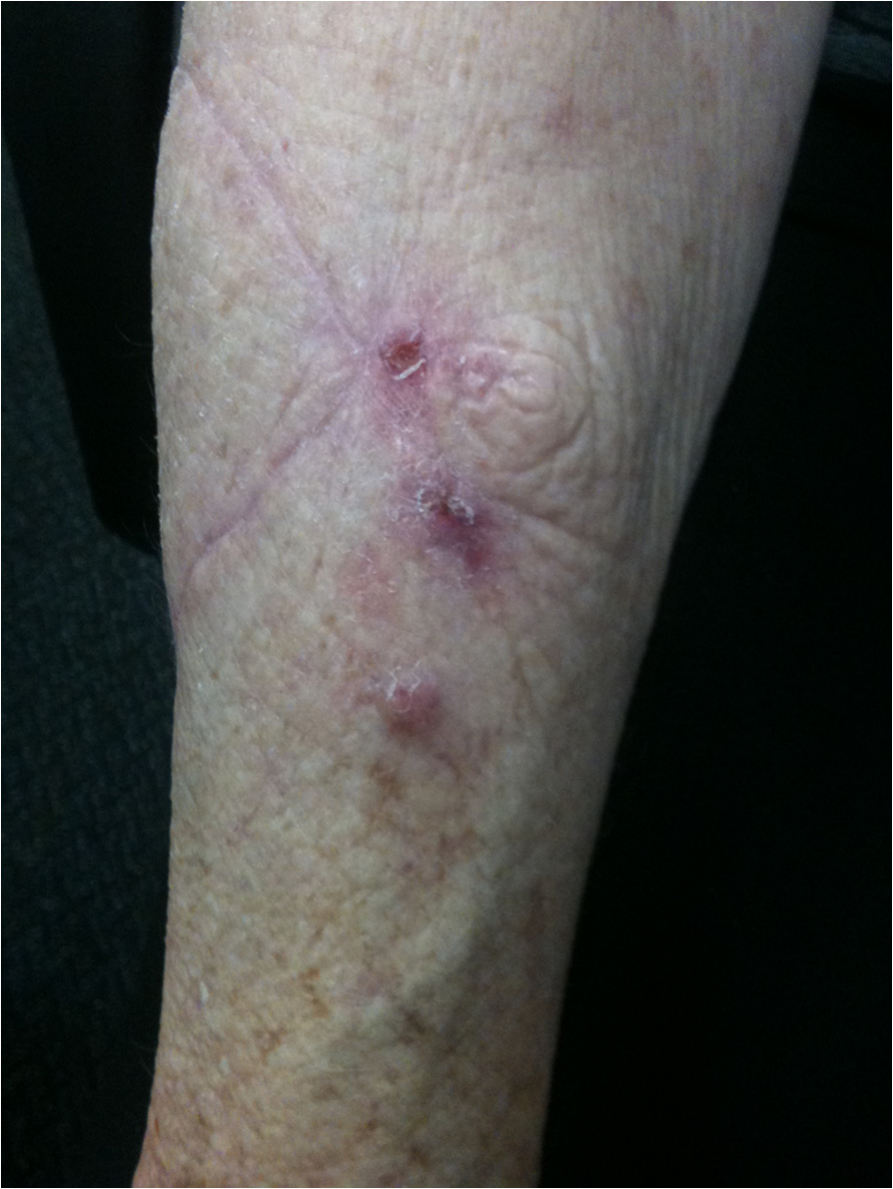
Local paradoxical reaction following complete surgical excision of original lesion (see surgical scar) manifest by the appearance of 3 new lesions within 3 cm of the surgical site 6 weeks after antibiotic commencement.

**Figure 2:**
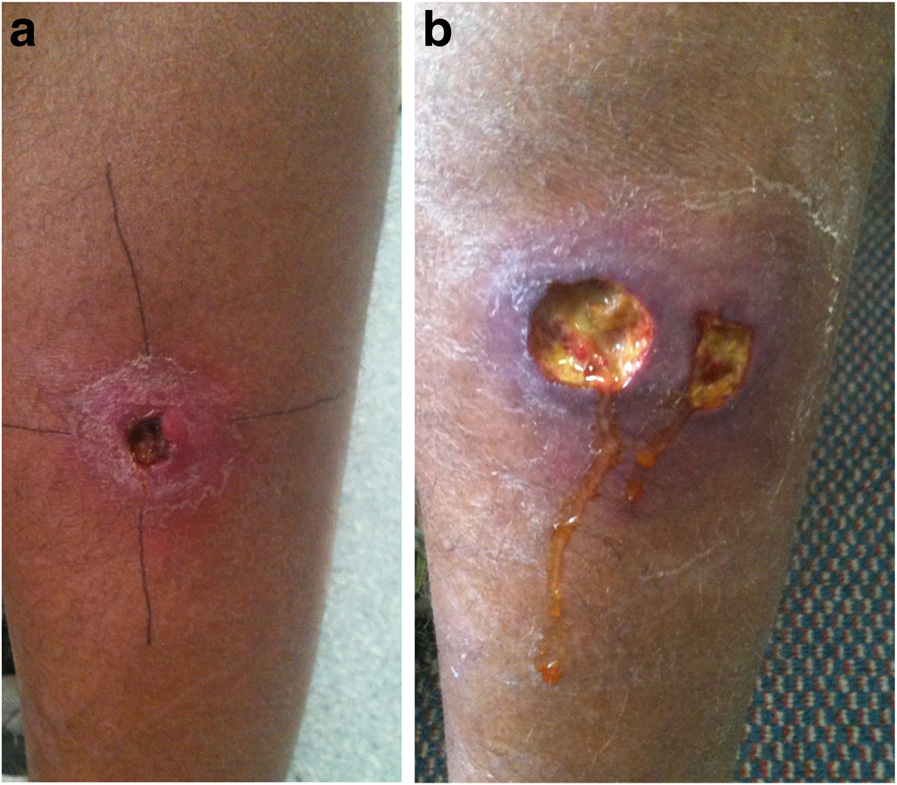
**Illustration of clinical features representing a wound paradoxical reaction.a-b**: Buruli ulcer lesion posterior calf at commencement of antibiotic treatment with induration 40x42 mm diameter **(a)**. Lesion after 5 weeks antibiotic treatment complicated by a paradoxical reaction manifest by increased induration around lesion (60x40 mm diameter), increased serous discharge and new ulceration adjacent to initial lesion **(b)**.

The site of the paradoxical lesion was defined as ‘wound’ if it occurred in wound margins or involved generalized induration around the original lesion, ‘local’ if it was separate to but within 3 cm of the initial lesion or ‘distant’ if it occurred greater than 3 cm from the initial lesion (Figures 1 and 2). A patient could have more than one ‘paradoxical episode’ if there were further new lesions separated by site or time from the original paradoxical lesion. The position of a *M. ulcerans* lesion was described as distal if it was on or below the elbow or knee. A ‘major excision’ involved the use of a split skin graft (SSG) or a vascularised skin and tissue flap to cover the defect. Positive margins were defined as the presence of granulomatous inflammation or necrotic tissue extending to one or more of the surgical excision margins on histopathological examination [16]. Immune suppression was defined as current treatment with immunosuppressive medication (e.g. prednisolone) or an active malignancy.

Drug dosages included rifampicin 10 mg/kg/day (up to a maximum of 600 mg daily), clarithromycin 7.5 mg/kg twice daily (up to a maximum of 500 mg twice daily), ciprofloxacin 500 mg twice daily, moxifloxacin 400 mg daily, and amikacin 15 mg/kg/day. Mycobacterial cultures were performed using Lowenstein–Jensen media and incubated for 12 weeks.

Data was analysed using Epi-Info 6 (CDC, Atlanta) and STATA 12 (StataCorp, Texas, USA). Proportions were compared using 2×2 tables and the Chi-squared test. A Kaplan-Meier curve was used to measure the cumulative incidence of first paradoxical reactions. Outcome data were censored at the time of a paradoxical reaction, disease recurrence or 12 months from antibiotic initiation.

A Poisson regression model was used to assess incidence rates and associations of variables with the first paradoxical episode in a patient. Crude rate ratios were determined by performing univariable analyses. A multivariable analysis was performed including the variables sex and age *a priori* and all variables showing strong evidence of an association with paradoxical reactions in the crude analysis (assessed by p ≤ 0.10). The variable ‘positive margins’ was not included in the multivariable model due to missing data (not all patients had surgical excisions).

### Ethics

This study was approved by the Barwon Health Research and Ethics Committee. Verbal patient consent was given for the collection and use of data. All data were analysed anonymously.

## Results

From 1/1/1998 until 29/12/2011, 160 patients received antibiotics at Barwon Health as treatment for *M. ulcerans*. One hundred and fifty-six had at least 12-months follow-up and were included in the study. Two patients died during treatment and two patients were lost to follow-up and were not included. Baseline characteristics of the study population are reported in Table 1. The median duration of antibiotic treatment was 58 days (IQR 42–90 days).

**Table 1:** Poisson regression model showing adjusted and unadjusted associations between identified factors and first paradoxical reactions rates

	**Number (%) in cohort**	**Number (%) experiencing a PR**	**Follow-up** **(years)**	**Rate per 100 person-years (95% CI)**	**Crude rate ratio (95% CI)**	p-value*	**Adjusted rate ratio (95% CI)**	**p-value#**
Sex								
Male	86 (55.1)	15 (17.4)	72.5	20.7 (12.5,34.3)	1	0.26	1	0.64
Female	70 (44.9)	17 (24.3)	55.0	30.8 (19.1,49.5)	1.48 (0.74,2.98)		0.83 (0.38,1.81)	
Age (years)								
0-<15	13 (8.3)	3 (23.1)	9.9	30.4 (9.8,94.2)	2.94 (0.74,11.76)	0.01	2.77 (0.67,11.38)	0.16
15-<60	62 (39.7)	6 (9.7)	58.1	10.3 (4.6,23.0)	1		1	
≥60	81 (51.9)	23 (28.4)	59.8	38.4 (25.5,57.9)	3.72 (1.51,9.14)		2.84 (1.12,7.17)	0.03
Lesion type								
Ulcer	137 (87.8)	24 (17.5)	116.0	20.7 (13.9,30.9)	1	0.03	1	
Nodule	10 (6.4)	4 (40.0)	6.3	63.3 (23.7,168.6)	3.06 (1.06,8.81)		1.98 (0.57,6.91)	0.29
Oedematous	9 (5.8)	4 (44.4)	5.4	73.9 (27.7,196.8)	3.57 (1.24,10.29)		3.44 (1.11,10.70)	0.03
Lesion site								
Upper limb	55 (35.3)	11 (20.0)	44.7	24.6 (13.9,44.4)	1			
Lower limb	99 (63.5)	21 (21.2)	80.3	25.9 (16.9,39.7)	1.05 (0.51,2.18)	0.60	-	-
Torso	2 (1.3)	0 (0.0)	-	-	-			
Lesion position								
Proximal	16 (10.3)	2 (12.5)	14.2	14.1 (3.5,56.5)	1	0.35	-	-
Distal	140 (89.7)	30 (21.3)	113.6	26.4 (18.5, 37.8)	1.87 ( 0.45,7.82)			
Over a joint								
No	91 (58.3)	17 (18.7)	76.2	22.5 (13.9,35.9)	1	0.45	-	-
Yes	65 (41.7)	15 (23.1)	51.5	29.1 (17.6,48.3)	1.31 (0.65,2.61)			
Positive margins								
No	36 (32.7)	4 (11.0)	32.5	12.3 (4.6,32.8)	1	0.03	-	-
Yes	74 (67.3)	20 (27.0)	56.5	35.4 (22.8,54.8)	2.87 (0.98,8.41)			
Any excision								
No	42 (27.0)	6 (14.3)	36.5	16.5 (7.4, 36.6)	1	0.20	-	-
Yes	114 (73.0)	26 (22.8)	91.3	28.5 (19.4,41.8)	1.72 (0.71,4.20)			
Major excision								
No	82 (52.6)	11 (13.4)	71.9	15.3 (8.5,27.6)	1	0.01	1	0.14
Yes	74 (47.4)	21 (28.4)	55.9	37.6 (24.5,57.6)	2.45 (1.18,5.09)		1.78 (0.82,3.87)	
Diabetes								
No	143 (91.7)	27 (18.9)	118.9	22.7 (15.6,33.1)	1	0.09	1	0.77
Yes	13 (8.3)	5 (38.5)	8.8	56.7 (23.6,136.2)	2.50 (0.96,6.49)		1.19 (0.37,3.89)	
Immune suppression								
No	145 (93.0)	28 (19.3)	120.0	23.3 (16.1,33.8)	1	0.18	-	-
Yes	11 (7.1)	4 (36.4)	7.7	51.7 (19.4,137.6)	2.21 (0.78,6.31)			
Rifampicin								
No	9 (5.8)	2 (22.2)	7.4	26.9 (6.7,107.5)	1	0.92	-	-
Yes	147 (94.2)	30 (20.4)	120.3	24.9 (17.4,35.7)	0.93 (0.22,3.88)			
Ciprofloxacin								
No	55 (35.3)	13 (23.6)	43.5	29.9 (17.4,51.5)	1	0.44	-	-
Yes	101 (64.7)	19 (18.8)	84.3	22.5 (14.5,35.7)	0.75 (0.37,1.53)			
Clarithromycin								
No	108 (69.2)	24 (22.2)	87.1	27.6 (18.5,41.1)	1	0.40	-	-
Yes	48 (30.8)	8 (16.7)	40.7	19.7 (9.8,39.3)	0.71 (0.32,1.59)			
Ethambutol								
No	145 (93.0)	28 (19.3)	120.1	23.3 (16.1,33.8)	1	0.17	-	-
Yes	11 (7.1)	4 (36.4)	7.6	52.3 (19.6,139.5)	2.25 (0.79,6.40)			
Amikacin								
No	151 (96.8)	28 (18.5)	126.0	22.2 (15.3,32.2)	1	<0.001	1	<0.01
Yes	5 (3.2)	4 (80.0)	1.8	228.6 (85.8,609.2)	10.29 (3.61,29.33)		6.33 (2.09,19.18)	
Moxifloxacin								
No	151 (96.8)	30 (19.9)	124.6	24.1 (16.8,34.4)	1		-	
Yes	5 (3.2)	2 (40.0)	3.1	64.0 (16.0,255.8)	2.66 (0.64,11.12)	0.24		-

*CI* confidence interval, *PR* paradoxical reaction, *likelihood ratio test, #Wald test.

### First paradoxical episode

**Figure 3:**
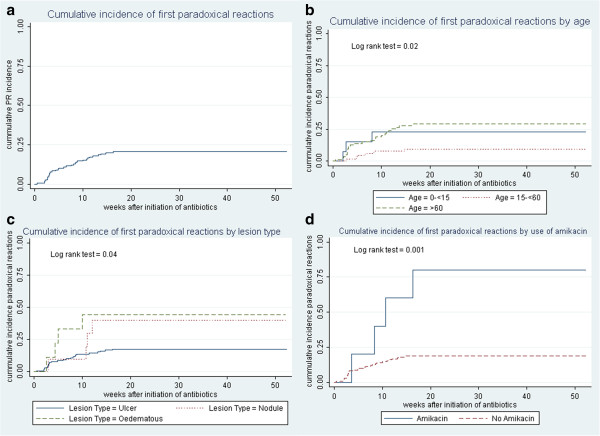
**Cumulative incidence of paradoxical reactions. a**: Cumulative incidence of first paradoxical reactions. **b**: Cumulative incidence of first paradoxical reactions by age. **c**: Cumulative incidence of first paradoxical reactions by lesion type. **d**: Cumulative incidence of first paradoxical reactions by use of amikacin.

**Figure 4:**
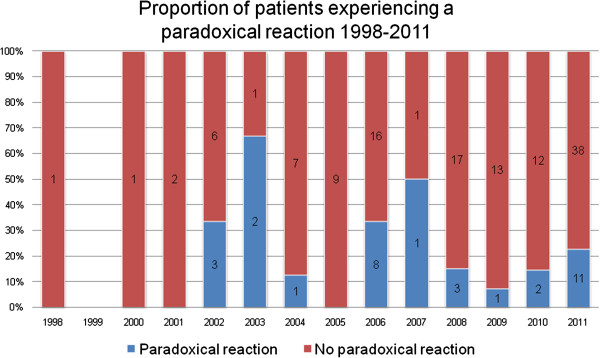
Number and proportion of patients developing paradoxical reactions per calendar year.

Thirty-two of 156 (21%) patients developed paradoxical reactions. Baseline characteristics for patients developing paradoxical reactions are shown in Table 1. Over a combined follow-up time of 127.8 years, the rate of first paradoxical reactions was 25.0 (95% CI 17.7–35.4) per 100 person-years. The cumulative incidence of first paradoxical reactions, also stratified by age, lesion type and use of amikacin are shown in Figure 3. The median time from antibiotic administration to development of the first reaction was 39 days (IQR 20–73 days). The number and proportion of patients developing paradoxical reactions per calendar year is shown in Figure 4.

### All paradoxical episodes

There were 42 paradoxical episodes. Twenty-six (81%) patients experienced one episode and 6 (19%) had multiple episodes (3 with 2 episodes, 2 with 3 episodes and 1 with 4 episodes). Of those with multiple episodes, four experienced episodes separated in time and two had simultaneous episodes separated by site. Thirty-two (76%) episodes occurred during antibiotic treatment. Ten (24%) episodes occurred a median of 37 days (range 16-150 days) after antibiotic treatment.

### Lesion site

For the 42 paradoxical episodes, the site of the reaction was wound in 23 (55%), local in 11 (26%) and distant in 8 (19%) episodes.

Eight patients developed new local lesions: for 3 patients this involved 1 lesion, in 4 patients 2 lesions, and in 1 patient 6 lesions. For those with multiple local lesions (5 patients), in 3 patients they occurred simultaneously and in 2 patients they appeared at separate times. Seven patients developed new distant lesions: three developed lesions on a non-contiguous body part; 2 of these involved single lesions on alternate limbs (one on the left foot with an original on the right foot, and one on the left lower leg with an original on the right arm) and one developed 2 lesions on the right buttock after an original lesion on the ipsilateral calf. The other 4 patients developed new lesions on an adjacent body part to that of the original lesion (e.g. calf to thigh).

### Diagnosis

The diagnosis of paradoxical reactions was made on clinical and histological criteria in 27 (84%) patients. For 5 (16%) patients clinical criteria alone were used, although in 3 (9%) of these cases it was supported by negative AFB examinations and negative mycobacterial cultures.

A mycobacterial culture was negative in 33/33 (100%) paradoxical episodes in 26 patients in which it was performed at a median of 59 days (IQR 21-80 days) after antibiotics commenced. All were performed ≥ 2 weeks after antibiotics commenced. *M. ulcerans* PCR was positive in 23/26 (88%) episodes in 21 patients and an AFB stain was positive in 23/39 (59%) episodes in 31 patients in which they were done.

### Treatment

Paradoxical reactions were managed without changing the regimen or duration of antibiotic administration in 22 (52%) episodes, antibiotics were prolonged in 10 (24%) episodes, antibiotics were added and prolonged in 9 episodes (21%) and an antibiotic added but not prolonged in 1 case (2%). Surgery was performed to manage paradoxical reactions in 31 (74%) episodes. This involved debridement in 10 (32%), excision and primary closure in 7 (23%), excision and SSG in 11 (35%), and excision and flap in 3 (10%) one of whom also had a SSG.

Post-February 2009, when paradoxical reactions were first recognized in our practice, compared to pre-February 2009, treatment involved more cases with no change in the antibiotic regimen or duration (12/15 compared with 11/27, OR 5.82, 95% CI 1.12–34.07, p = 0.02) and more cases where no further surgery was performed (9/15 compared with 2/27, OR 18.75, 95% CI 2.62–172.73, p < 0.001). There was a trend to less reconstructive surgery (SSG or flap) performed in the post-February 2009 period (3/15 compared with 11/27, OR 0.36, 95% CI 0.06–1.90, p = 0.18).

### Severe paradoxical episodes

Nine of 42 (21%) cases were clinically severe; 3 occurred prior to prednisolone use and resulted in significant tissue destruction, further extensive surgery and antibiotic changes. For example, from an initial lesion on the right hand, a patient developed severe inflammation and necrosis of the hand, fingers and forearm, including tendons, requiring extensive debridement with a free tissue flap reconstruction and antibiotics prolonged until 116 days. Prednisolone was introduced into our practice to treat severe reactions in June 2010 [17]. Following this 6 severe cases received prednisone at a dose of 0.5-1 mg/kg daily for 4-6 weeks with marked clinical improvement in lesion appearances within days to weeks. Only 2 patients required minimal debridement of their lesions after prednisolone commencement and the antimicrobial regimen was not changed nor prolonged beyond 12 weeks.

### Outcomes

All patients with paradoxical lesions experienced healing of their lesions without disease recurrence after 12 months follow-up from antibiotic commencement.

### Predictors of paradoxical reactions

On univariate analyses the following variables were strongly associated with the development of paradoxical reactions: age (p = 0.01), lesion type (p = 0.03), major excision (p = 0.01), positive margins (p = 0.03) and the use of amikacin (p < 0.001) in the initial antibiotic regimen. Diabetes (p = 0.09) showed weaker evidence for an association (Table 1). On multivariable analysis adjusting for age, sex, lesion type, major excision, diabetes and the use of amikacin in the initial antibiotic regimen, age ≥ 60 years (RR 2.84, 95% CI 1.12-7.17, p = 0.03), an oedematous lesion (RR 3.44, 95% CI 1.11-10.70, p = 0.03) and the use of amikacin in the initial antibiotic regimen (RR 6.33, 95% CI 2.09-19.18, p < 0.01) remained strongly associated with the development of paradoxical reactions (Table 1).

## Discussion

This observational study of a large patient cohort treated with antibiotics for *M. ulcerans* provides the first comprehensive descriptions of the incidence, clinical spectrum, diagnostic features (microbiological and PCR results), treatments and outcomes of paradoxical reactions occurring following antibiotic treatment in an Australian population. It demonstrates that paradoxical reactions were common, occurring in approximately one in five patients. They occurred anywhere from the first week of treatment until 8 months post antibiotic commencement, although most occurred within 3–10 weeks of antibiotic initiation. At least one fifth of reactions occurred after completion of antibiotics, reinforcing other recent evidence from Africa that new clinical symptoms post antibiotic treatment should not be assumed to be treatment failure and recurrent infection [7]. Rates of paradoxical reactions in *M. ulcerans* treated patients are not well described, but a rate of 23% was recently described in a cohort from Africa [8]. Interestingly the rate in our study is similar to the 16% of HIV patients beginning antiretroviral treatment who develop immune reconstitution disease [18]. In our earlier experience from a smaller cohort (90 patients) we described paradoxical reactions occurring in 8 (9%) patients [19].

Approximately half the reactions involved an increase in induration surrounding a lesion, often with increased amounts of wound discharge. However in nearly one-fifth of cases, the clinical presentation included the development of lesions that were distant from the initial site, including alternate limbs or body parts. These likely represent subclinical sites of infection that become evident secondary to reversal of *M. ulcerans* induced immune inhibition upon commencing antibiotics. Reactions could involve multiple simultaneous lesions, and multiple paradoxical episodes can also occur in the same patient, separated either in site or time.

Although a standard definition of paradoxical reactions has not been developed our study provides information that may be useful in distinguishing them from antibiotic treatment failure. In our experience mycobacterial cultures were always negative in patients experiencing a paradoxical reaction ≥ 2 weeks after antibiotic commencement. Likewise cultures were negative in paradoxical lesions recently described by others [7,9]. Conversely both AFB stains and PCR tests were usually positive, likely from detection of non-viable *M. ulcerans,* meaning that when positive they are not useful in distinguishing between these two treatment outcomes.

**Figure 5:**
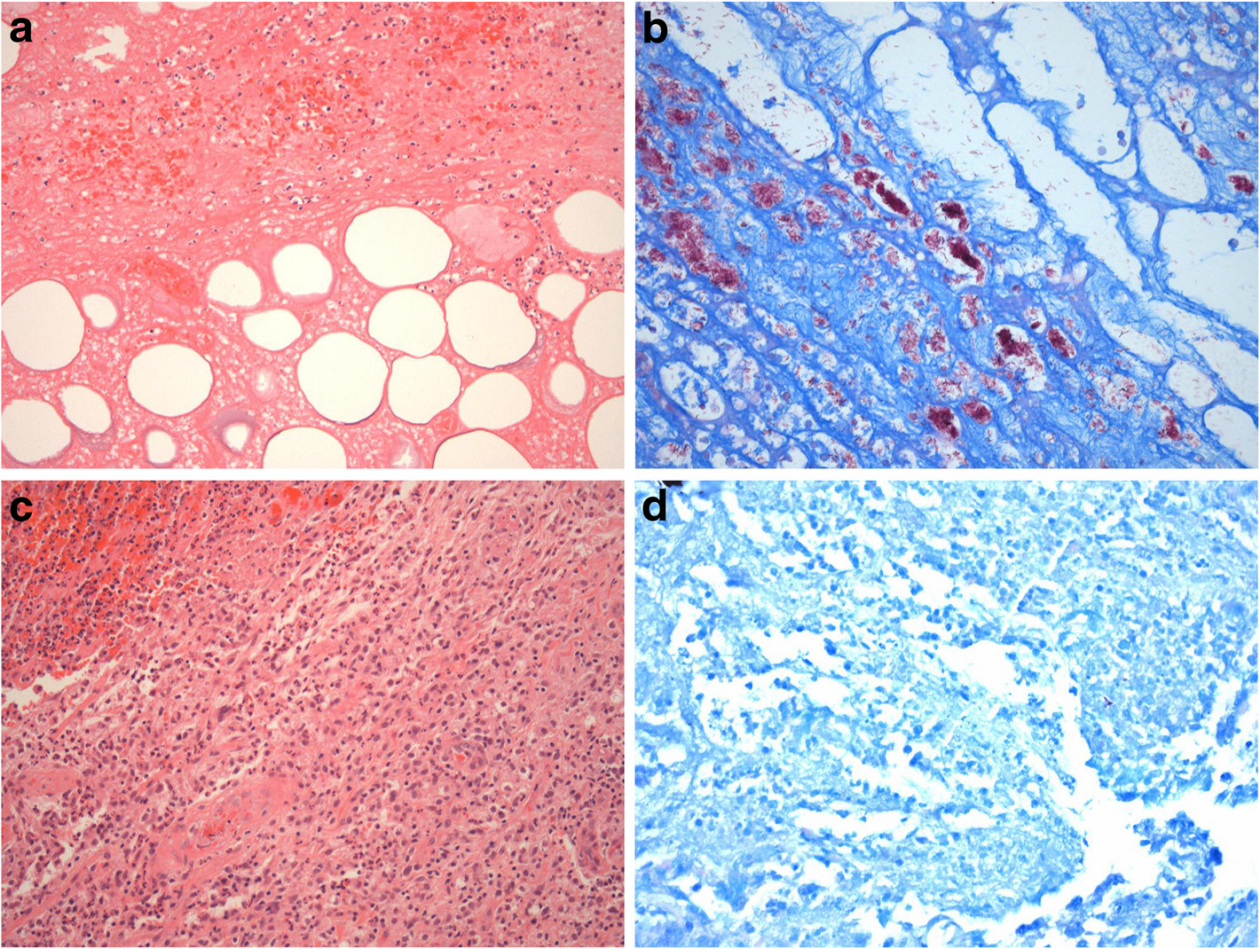
**Illustration of histological features representing a paradoxical reaction. a-d**: Initial lesion before antibiotic treatment showing a sparse acute inflammatory reaction around necrotic fat and subcutaneous tissue **(a)** with high numbers of extracellular acid-fast bacilli (in pink) on Wade-Fite stain **(b)**. After 8 weeks of antibiotic treatment showing a paradoxical reaction manifest by a dense inflammatory reaction including multinucleated giant cells **(c)** and no extracellular acid-fast bacilli seen with Wade-Fite stain **(d)**.

It is critically important to distinguish between paradoxical reactions and treatment failure potentially related to issues such as sub-optimal medication adherence or drug resistance. We believe that the clinical evolution of lesions, supported by consistent histopathological findings in most cases (Figure 5), as well as negative mycobacterial culture results, strongly suggest that cases in our study represent true paradoxical reactions and not progressive disease secondary to antibiotic failure. This includes 5 patients previously considered to have antibiotic treatment failures, [16] who were retrospectively determined in this study to have instead experienced paradoxical reactions. In these cases the histological appearances of excised involved tissue showed evidence of paradoxical reactions [6,15] and differed markedly from the initial lesions which showed classical appearances of untreated *M. ulcerans* infections [2]. It is likely that patients were similarly previously misclassified in other studies leading to incorrect conclusions that antibiotics were ineffective [20,21]. We found no evidence of bacterial super-infection causing the ‘paradoxical’ lesions in our study: histopathology of excised lesions showed no evidence of it and all reactions settled without specific antibiotic treatment for it.

The recognition of paradoxical reactions has led to a change in the way *M. ulcerans* infections are managed in our practice. It has allowed a significant reduction in the frequency of cases managed with surgical intervention (reduced by 19 times) and in the frequency of the change or prolongation of antibiotic regimens (reduced by 6 times). There also appears likely to have been a decrease in the amount of reconstructive surgery performed, although due to the low numbers of cases, the strength of this association was not strong enough to definitively make this conclusion.

Severe paradoxical reactions can have significant adverse consequences – a number of patients required major surgical debridements, often on multiple occasions and requiring reconstructive surgery, resulting in significant patient morbidity and cost [22]. However we observed that for severe cases prednisolone effectively controlled the reaction, preserved tissue grafts and avoided further major surgery without adverse consequences such as the need to significantly prolong antibiotics or the development of treatment failure [17].

Our current management recommendation is that when a paradoxical reaction is suspected, where possible, a specimen should be sent for urgent histopathological examination to confirm its presence initially, with mycobacterial cultures performed for later confirmation. In settings where these procedures are not available, if the clinical appearance is consistent, and other causes such as suboptimal medication adherence are excluded, then empirical management for paradoxical reactions would be a reasonable approach. However there is an urgent need to develop new diagnostic tests to aid the distinction between paradoxical reactions and treatment failure that are feasible, affordable and available in resource-limited settings where most of the infections are treated. For mild cases we would recommend that most can be managed with observation alone, hence avoiding further surgery or a change in antibiotics. If necessary limited debridement without reconstructive surgery is preferred. In severe episodes a course of prednisolone (0.5–1.0 mg/kg daily tapered over 4–8 weeks) and prolongation of antibiotics to a total of 12 weeks treatment can be considered. Further research is urgently required to validate these recommendations in other settings. Currently paradoxical reaction risk has not been used in our practice as an indication to avoid antibiotics and treat with surgical excision alone.

To our knowledge, this is the first published study to examine factors associated with the development of paradoxical reactions. The incidence appeared to increase in older adults and children; those greater than 60 years had a nearly three times increased rate compared to younger adults. Children less than 15 years had a similar increased rate, but limited by low numbers of children in the study this association was less strong (p = 0.16). As T-cell mediated immunity is felt to play a vital role in host immunity against *M. ulcerans*, [23,24] possibly the organism has a more powerful inhibitory effect against this in older adults and children due to aging or less developed immune systems respectively. This may allow the organism to replicate more freely resulting in an increased organism load and secondary antigenic stimulus, or allow a greater potential for rapid immune function improvements when the inhibitory effects of mycolactone toxin are removed with antibiotics [13,14].

Oedematous lesions are less common, [1] but are often severe and rapidly progressive, involving large areas of the body, and frequently leading to extensive tissue damage [24]. A three times increased rate of paradoxical reactions for oedematous lesions was demonstrated in our study and may result from an increased antigen load due to the large burden of mycobacteria usually present in these lesions [24]. In addition, it is probable there has been a poor initial immune response to the infection allowing it to spread, and once this is aided by the use of antibiotics an aggressive immune response to the large antigen load may result. This may be similar to the recognized increase rate of paradoxical reactions in HIV-infected people commencing antiretroviral therapy in those with more extensive or disseminated opportunistic infections [18], including mycobacterium tuberculosis [25], thought to be due to increased antigen burden.

Amikacin was associated with greater than a 6 times increased rate of paradoxical reactions. Amikacin is highly bactericidal *in vitro*[26] and this rapid killing of organisms may act as a strong stimulus for the development of paradoxical reactions, since the killing of micro-organisms likely underlies its pathogenesis [15]. Additionally, it may be that the combination of rifampicin with amikacin is responsible for the stimulus, as this combination is more bactericidal than amikacin alone in mouse models [26].

In our study the most commonly used orally administered companion drugs to rifampicin (ciprofloxacin and clarithromycin) were not associated with an increased rate of paradoxical reactions which may relate to their known anti-inflammatory properties [27,28]. These findings may offer further advantages to using these antibiotics to obtain fully orally administered regimens for *M. ulcerans* treatment [19,29]. Furthermore, those who had antibiotics alone compared to antibiotics plus surgery were not at an increased risk of paradoxical reactions.

It is known that there is a higher risk of paradoxical reactions in HIV-infected people commencing antiretroviral therapy with increasing levels of immunosuppression at baseline [18]. In our study on univariable analysis the rate of paradoxical reactions was double in those who were immunosuppressed (RR 2.21, 95%CI 0.78–6.31), but the strength of the association was weakened (p = 0.18) by the small numbers of immunosuppressed patients in the cohort (n = 11, 7.1%). Hence further study with larger cohorts is recommended to further clarify this association.

There are limitations to our study. Firstly, there are no standardized definitions for paradoxical reactions, and thus there may be inaccuracies in case ascertainment. However with 84% of cases confirmed on histology we feel that our cases accurately represent paradoxical reactions. Secondly, due to its observational design there may be other unmeasured confounders not taken into account in the analysis of associations that could potentially affect the validity of the findings. Observed associations should be further studied in prospective trials. Thirdly there was no data on the size of lesions and therefore we could not measure the effect of lesion size on the rate of paradoxical reactions. However, the type of surgery approximately separates small and large lesions, as small lesions are amenable to excision and primary closure whereas larger lesions require SSG or vascularised flaps (major surgery) to achieve tissue closure, and this information was included in the analyses.

## Conclusions

Paradoxical reactions occur frequently during or after antibiotic treatment of *M. ulcerans* lesions. They mostly involve changes around the initial infection site but can be distantly located, and lesions can be multiple with episodes separated in both site and time. Mycobacterial cultures are usually negative, but PCR and AFB examinations of involved tissue usually remain positive. Paradoxical reactions may be increased in older adults, oedematous forms of disease, and in those treated with amikacin. Recognition of paradoxical reactions led to changes in management with less surgery, fewer antibiotic modifications and use of prednisolone for severe reactions.

## Abbreviations

AFB: Acid fast bacilli; PCR: Polymerase chain reaction; SSG: Split skin graft; IQR: Interquartile range.

## Competing interests

The authors declare that they have no competing interests.

## Authors’ contributions

DPOB: Designed the study, collected and analysed the data, and wrote the initial draft of the manuscript. MR: Reviewed the histopathology and helped to draft the manuscript. NDF: Helped collect the data and helped to draft the manuscript. AW: Helped collect the data and helped to draft the manuscript. AMcD: Helped collect the data and helped to draft the manuscript. PC: Helped collect the data and helped to draft the manuscript. AH: Helped collect the data and helped to draft the manuscript. RR: Helped collect the data and helped to draft the manuscript. EA: Helped collect the data and helped to draft the manuscript. All authors read and approved the final manuscript.

## Pre-publication history

The pre-publication history for this paper can be accessed here:

http://www.biomedcentral.com/1471-2334/13/416/prepub
